# Machine learning-based selection of immune cell markers in osteosarcoma: prognostic determination and validation of CLK1 in disease progression

**DOI:** 10.3389/fimmu.2024.1468875

**Published:** 2024-12-17

**Authors:** Nan Zhang, Zhou Haizhen, Runqi Zhang, Xiaoju Li

**Affiliations:** ^1^ Department of Pathology, Honghui Hospital, Xi’an Jiaotong University College of Medicine, Xi’an, Shaanxi, China; ^2^ Department of Orthopedics, Honghui Hospital, Xi’an Jiaotong University College of Medicine, Xi’an, Shaanxi, China; ^3^ Pathology Teaching and Research Office, Xi’an Medicine College, Xi’an, Shaanxi, China

**Keywords:** osteosarcoma, intratumor heterogeneity, prognosis, immunotherapy, immune cell markers

## Abstract

**Introduction:**

Osteosarcoma (OS) is a malignancy of the bone that mainly afflicts younger individuals. Despite existing treatment approaches, patients with metastatic or recurrent disease generally face poor prognoses. A greater understanding of the tumor microenvironment (TME) is critical for enhancing outcomes in OS patients.

**Methods:**

The clinical and RNA expression data of OS patients were extracted from the TARGET database. The single-cell RNA sequencing (scRNA-seq) data of 11 OS samples was retrieved from the GEO database, and analyzed using the Seurat package of R software. Copy number variation (CNV) was analyzed using the InferCNV software. The potential interactions between the different cells in the TME was analyzed with the CellChat package. A multi-algorithm-based computing framework was used to calculate the tumor-infiltrating immune cell (TIIC) scores. A prognostic model was constructed using 20 machine learning algorithms. Maftools R package was used to characterize the genomic variation landscapes in the patient groups stratified by TIIC score. The human OS cell lines MG63 and U2OS were used for the functional assays. Cell proliferation and migration were analyzed by the EdU assay and Transwell assay respectively. CLK1 protein expression was measured by immunoblotting.

**Results:**

We observed higher CNV in the OS cells compared to endothelial cells. In addition, there was distinct transcriptional heterogeneity across the OS cells, and cluster 1 was identified as the terminal differentiation state. S100A1, TMSB4X, and SLPI were the three most significantly altered genes along with the pseudo-time trajectory. Cell communication analysis revealed an intricate network between S100A1+ tumor cells and other TME cells. Cluster 1 exhibited significantly higher aggressiveness features, which correlated with worse clinical outcomes. A prognostic model was developed based on TIIC-related genes that were screened using machine learning algorithms, and validated in multiple datasets. Higher TIIC signature score was associated with lower cytotoxic immune cell infiltration and generally inferior immune response and survival rate. Moreover, TIIC signature score was further validated in the datasets of other cancers. CLK1 was identified as a potential oncogene that promotes the proliferation and migration OS cells.

**Conclusion:**

A TIIC-based gene signature was developed that effectively predicted the prognosis of OS patients, and was significantly associated with immune infiltration and immune response. Moreover, CLK1 was identified as an oncogene and potential therapeutic target for OS.

## 1 Introduction

Osteosarcoma (OS) is the most common bone malignancy, and accounts for over 50% of bone sarcoma cases (1). It predominantly affects the long bones and is characterized by the *de novo* formation of osteoid tissues (2). Most patients are affected at a relatively younger age (3). OS is currently managed through adjuvant chemotherapy and surgery. Nevertheless, the 5-year survival rate for OS patients with metastases is lower than 20% (4). Given the challenges and limitations in the current treatment strategies for OS, there is a crucial need to identify new therapeutic targets that can enhance clinical efficacy and improve patient survival.

There has been an increasing focus on the tumor microenvironment (TME) for developing novel treatment strategies against cancer (5). The TME includes malignant cells, stromal cells, and the extracellular matrix (6), and plays a key role in tumor growth, metastasis, immune escape, and therapy resistance (7–10). In fact, the microenvironment of OS has been identified as a key determinant of patient prognosis (11). The stromal cells in the tumor tissues, particularly cancer-associated fibroblasts, directly contribute to immunosuppression (12). Numerous studies have developed TME-based models using machine learning approaches to predict prognosis and the response to immunotherapy (13–15). In this study, we utilized machine learning algorithms to establish a gene signature based on tumor-infiltrating immune cells (TIIC) for the prognostic stratification of OS patients.

The efficacy of the model was validated in multiple datasets. We also found that higher TIIC score was associated with significantly lower infiltration of cytotoxic immune cells. In other cancer types, a lower tumor immune infiltration signature score correlated with a better immune response and survival rate. Moreover, we identified CLK1 as an important factor in OS development and a potential therapeutic target.

## 2 Methods

### 2.1 Acquisition of transcriptomic data

The clinical and transcriptomic data of 85 OS patients were retrieved from the TARGET database. In addition, the microarray chip data of OS samples were obtained from the GEO database, including the GSE16091 (n=34), GSE21257 (n=53), and GSE39055 (n=37) datasets. The normalizeBetweenArrays function of the limma package was used to correct the chip data.

### 2.2 Acquisition of scRNA-seq data

The single-cell RNA sequencing (scRNA-seq) data of 11 OS samples was downloaded from the GEO database (GSE152048 dataset). Batch effects were addressed using the harmony method. Dimensionality reduction was performed using UMAP and t-SNE, as well as the Louvain clustering algorithm through the Seurat package.

### 2.3 Cell annotation

The immune cell clusters were separated using Sc-Type software for automatic annotation.

### 2.4 CNV and pseudo-time analysis of OS cells

The CNVs of tumor/OS cell subsets were analyzed using the InferCNV software with endothelial cells as a reference, and the CNVscore of each subgroup was calculated. Pseudo-time analysis of OS cell subsets was conducted using monocle2 software. Dimensionality reduction was performed using the DDRTree algorithm with default parameters to capture the cell differentiation process.

### 2.5 Intercellular communication analysis

The CellChat package was used to assess potential intercellular communication. The normalized gene expression matrix was imported using the CellChat function to create the CellChat object. The data was preprocessed using multiple functions.

### 2.6 Functional annotation of TIIC signature score

The acquisition of TIIC-related genes and cell annotation have been described in the additional file 1. The immune infiltrating cells were quantified using the tumor immune estimation resource (TIMER) algorithm (6 immune cells), ssGSEA algorithm (28 immune cells), MCPcounter algorithm (10 immune cells), and expression data ESTIMATE algorithm. Gene-set variation analysis (GSVA) and gene-set enrichment analysis (GSEA) were conducted to identify the GO terms and KEGG pathways. Enrichment analysis was performed using Metascape. GSVA was also performed to quantify 114 metabolic pathways from previous literature.

### 2.7 Identification and functional annotation of differentially expressed genes

The differentially expressed genes (DEGs) between the TIIC groups were screened using the limma package, with a screening threshold of P<0.05. The upregulated genes were subjected to GSEA using the clusterProfiler package. The gene sets related to KEGG and GOBP were enriched from the MSigDB database. The enrichment plot package was utilized for visualization when the BH corrected p-value was < 0.05.

### 2.8 Mutation analysis

The ‘maftools’ package was used to evaluate the difference in mutation load between the two groups and generate waterfall plots. The genes with differential mutation frequencies between the two groups were analyzed by the chi-square test. The CNV results were visualized using the ‘ggplot2’ package.

### 2.9 Development of TIIC-related risk signature

The candidate prognostic TIIC-related genes were screened through univariate Cox proportional hazard regression analysis. The significance of these genes was evaluated using three machine learning classification algorithms - random survival forest (RSF), least absolute shrinkage and selection operator regularized Cox regression (LassoCox), and Cox model based on possibility enhancement (CoxBoost). Furthermore, 20 machine learning algorithms were used for scoring, including RSF, conditional random forest (CForest), LassoCox, elastic net regression (Enet), Ridge regression, gradient boosting using regression tree (BlackBoost), parametric survival model regression (SurvReg), conditional inference tree (CTree), Cox proportional hazards model (CoxPH), ObliqueRSF, StepwiseCox, SurvivalSVM, generalized boosting regression model (GBM), Ranger, Cox model, and partial least squares regression of related technologies (PlsRcox). The most reliable model was selected on the basis of the comprehensive C index. The TIIC signature score based on the prognostic genes was developed using the RSF algorithm.

### 2.10 Cell culture

The human OS cell lines MG63 and U2OS were obtained from Procell Life Science and Technology Co., Ltd (Wuhan, China). The cells were cultured in MEM medium supplemented with 10% fetal bovine serum (FBS; Procell, Wuhan, China) and maintained at 37°C in an incubator with 5% CO_2_. The cell lines were transfected with CLK1-specific siRNAs or CLK1 overexpression vectors using Lipofectamine 3000 (Invitrogen, Carlsbad, CA, USA) as per the instructions. The medium was discarded 24 h later, and the cells were harvested and cultured overnight till confluency for the subsequent experiments.

### 2.11 EDU incorporation assay

Cell proliferation was assessed using the EdU (5-ethynyl-2’-deoxyuridine) assay kit (Beyotime Biotechnology) according to the manufacturer’s instructions. Briefly, the OS cells were seeded in 24-well plates at the density of 1 × 10^5 cells/well in complete medium. After incubating for 24 h, the cells were fixed with 4% paraformaldehyde for 15 minutes at room temperature, and then permeabilized with 0.3% Triton X-100 in phosphate-buffered saline (PBS) for 10 minutes. The cells were incubated with the EdU labeling solution as per the kit instructions, washed with PBS to remove excess EdU, and counterstained with DAPI (4’,6-diamidino-2-phenylindole) to stain the DNA. The stained cells were observed under a fluorescence microscope (Olympus or similar) using the FITC channel for EdU.

### 2.12 Colony formation assay

The suitably treated OS cells were seeded in 6-well plates and incubated at 36.7°C for 9 days. The cells were fixed and stained, and the colonies were counted.

### 2.13 Transwell assay

The suitably treated OS cells were seeded in the upper chambers of a Transwell insert (Corning, USA) in serum-free medium, and the lower chambers were filled with complete medium (with 10% FBS). Following overnight incubation, the cells adhering to the inner surface of the Transwell membrane were carefully removed, and those that migrated to the lower surface were fixed, stained with 0.5% crystal violet solution, and counted under a light microscope.

### 2.14 Immunoblotting

The cell lysates were heated at 96°C in 5× SDS loading buffer for 12 minutes. The denatured proteins were separated through SDS-PAGE and then transferred onto PVDF membranes (Millipore, USA). After blocking with 4% non-fat milk for 48 minutes, the membranes were incubated with primary antibodies specific for CLK1 (20439-1-AP, Proteintech, 1:1000) and ACTIN (81115-1-RR, Proteintech, 1:10000).

## 3 Results

### 3.1 Single-cell expression profiling of OS

**Figure 1:**
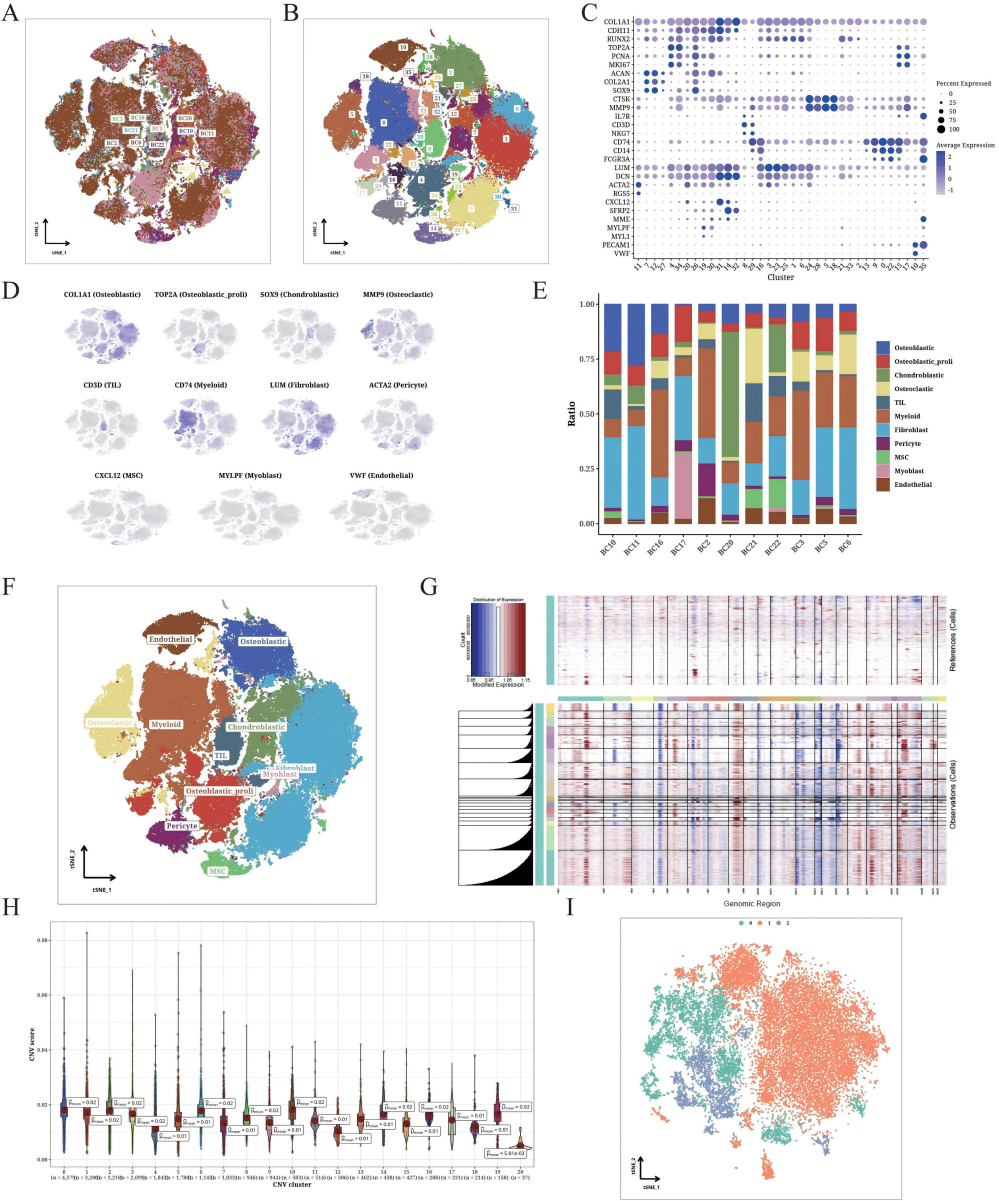
Detailed classification of OS cells. **(A)** t-SNE plot displaying the origin of the sorted OS cells. The different samples are color-coded. **(B)** t-SNE plot displaying the OS populations. The different clusters are color-coded. **(C)** Correlation matrix of marker gene expression in each cell cluster. **(D)** t-SNE plot showing unique expression of different marker genes in each annotated cluster. **(E)** Bar plot indicating the composition of different cell types from individual patients. **(F)** t-SNE plot illustrating the annotation results of cell types. **(G)** CNV heatmap of OS cells (endothelial cells as the internal reference). **(H)** CNV scores of different OS cell clusters. **(I)** t-SNE plot displaying the classification of identified OS cells based on CNV scores.

The scRNA-seq data of the OS samples exhibited a stable and similar cell distribution with low batch effects (**Figure 1A**). Using the t-SNE algorithm, we classified all cells into 36 clusters (**Figure 1B**). The expression pattern of the marker genes of each cell type are shown in **Figures 1C** and **D**, and the distribution of 11 cell types across the OS samples is shown in **Figure 1E**. We detected OS cells, tumor-infiltrating lymphocytes (TILs) and fibroblasts in all samples, along with an overall high abundance of myeloid cells (**Figure 1F**). The heat map shows the CNV scores of OS cells with endothelial cells as reference (**Figure 1G**). Furthermore, the OS cells had higher CNV scores compared to the endothelial cells. In particular, the clusters 0, 1, 2, 3, 6, 8, 10, 14, 16, and 19 had increased CNV, and clusters 4, 12, 18, and 20 showed decreased CNV (**Figures 1G, H**). The OS cells in these clusters were further divided into three subclusters (0–2) using t-SNE dimensionality reduction (**Figure 1I**).

### 3.2 Distinct trajectories of OS cells

**Figure 2:**
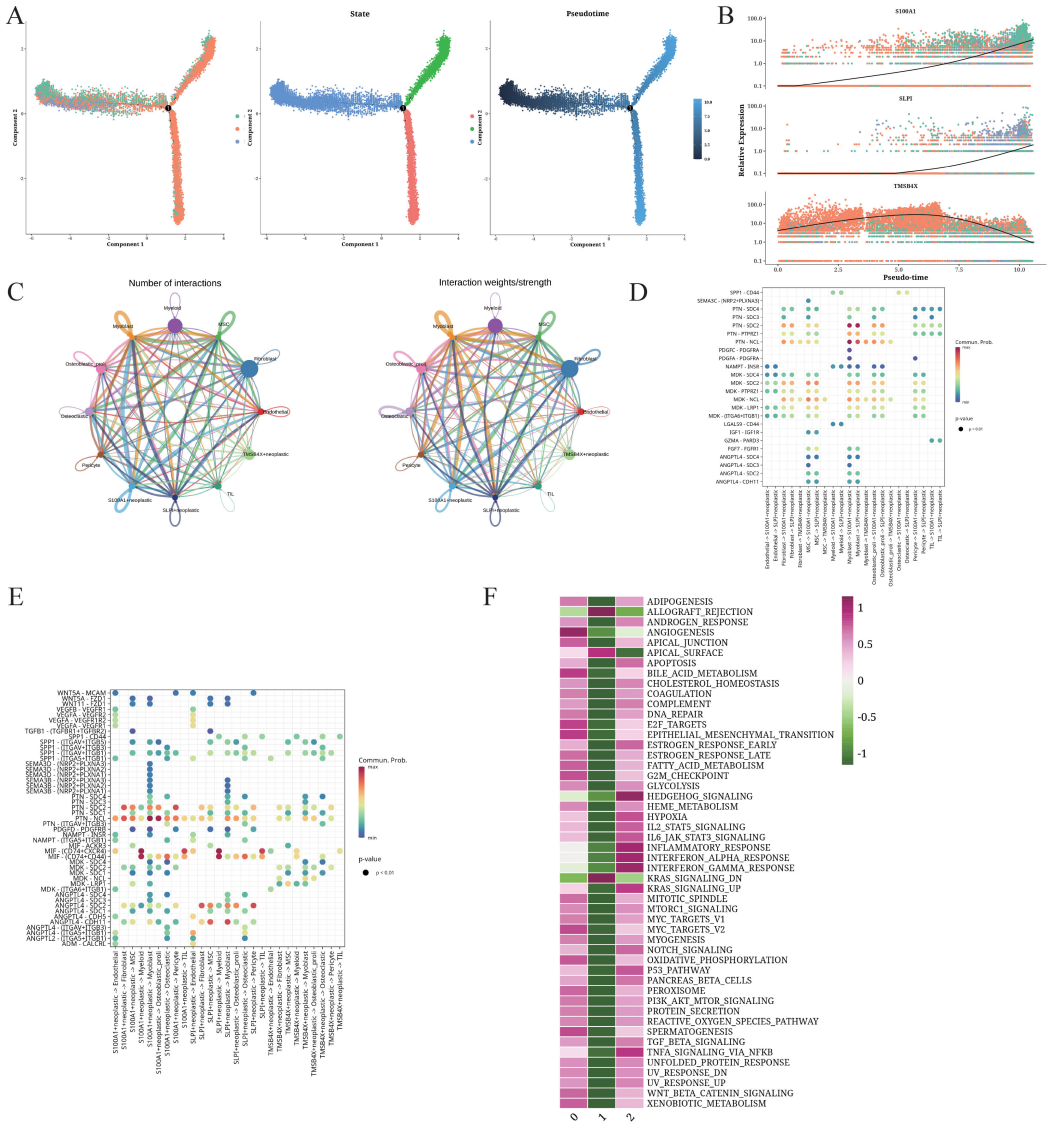
Trajectory and intercellular communication analysis of OS cells. **(A)** Differentiation trajectories, pseudo-time distribution, cell cluster distribution along pseudo-time, and the proportion of each cluster for all OS cells. **(B)** Relative alteration in the expression of S100A1, SLP1, and TMSB4X along pseudo-time. **(C)** Quantity and strength of intercellular communication between S100A1+ OS cells, TMSB4X+ OS cells, SLPI+ OS cells, and other cell types. **(D)** Bubble plot illustrating the interaction between S100A1+ OS cells, TMSB4X+ OS cells, SLPI+ OS cells, and the different cell ligands and receptors. **(E)** Bubble plot illustrating the interaction between different cell types and S100A1+ OS cells, TMSB4X+ OS cells, SLPI+ OS cells. **(F)** Enrichment analysis of S100A1+ OS cells, TMSB4X+ OS cells, SLPI+ OS cells.

The transcriptional heterogeneity of the OS cells was determined through trajectory analysis (**Figure 2A**). The pseudo-time progression showed that the clusters 0 and 2 spread throughout the entire trajectory, while cluster 1 was at the end of 2 branches. As shown in **Figure 2B**, S100A1, TMSB4X, and SLPI were the three most significantly altered genes along the pseudo-time trajectory. S100A1 and SLP1 were upregulated with the increase in pseudo-time value. The number of interactions between S100A1+ cells (cluster 0), TMSB4X+ cells (cluster 1), SLPI+ cells (cluster 2), and other cell types, and the intensity of communication are depicted in **Figure 2C**. The S100A1+ tumor cells in particular showed strong interaction with other TME cells. We also analyzed the ligand-receptor interactions between the different cells, and found that the S100A1+ tumor cells interacted with other cell types through the MDK-NCL receptor-ligand pair (**Figures 2D, E**). The clusters 0 and 2 were enriched in multiple biological processes across various pathways, while cluster 1 showed enrichment in the KRAS_SIGNALING_DN and ALLOGRAFT_REJECTION pathways (**Figure 2F**), which may be associated with immune response.

### 3.3 Transcription factor analysis of OS cells

**Figure 3:**
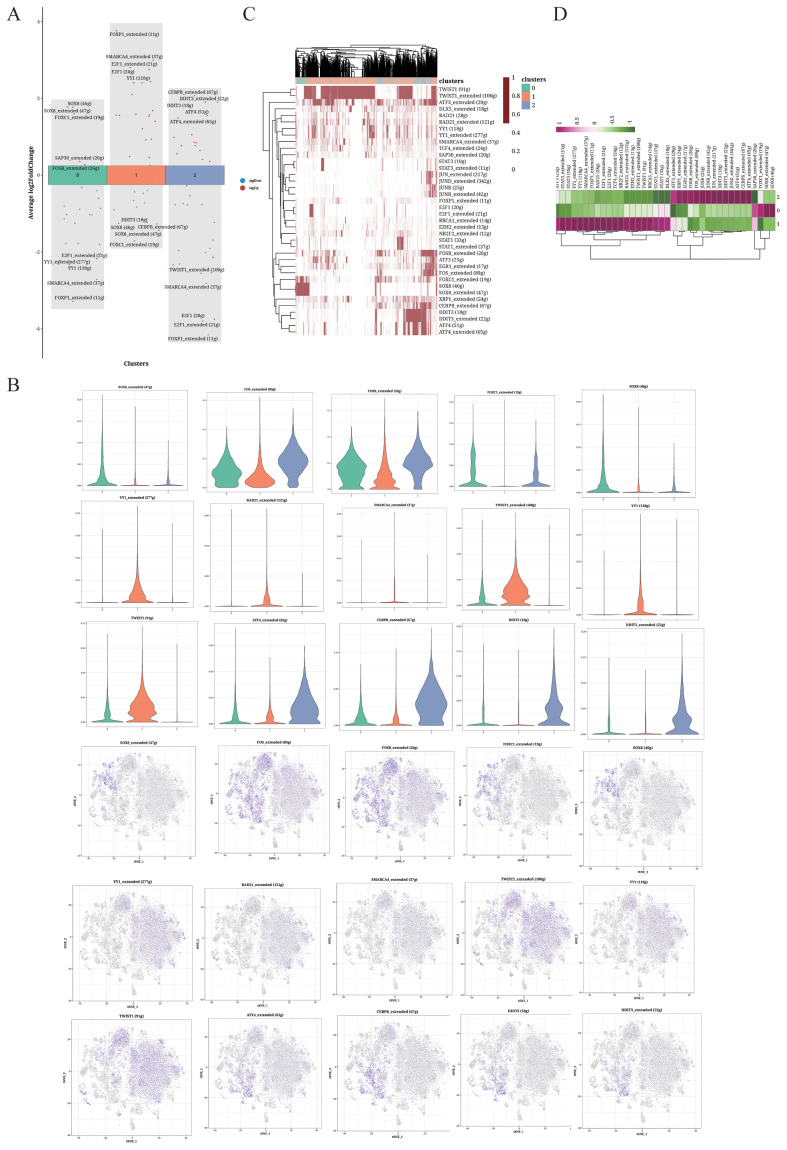
TF analysis of OS cells. **(A)** Volcano plot showing the top 5 highly expressed genes in each cluster. **(B)** Violin plots and UMAP plots of the top 5 upregulated genes. **(C, D)** Heatmaps displaying the distribution of TFs in the different clusters.

The differentially expressed transcription factors in each cluster are shown in **Figure 3A**. Cluster 1 was characterized by the upregulation of YY1, E2F and FOXP1, while cluster 1 showed high SOX8 expression. The expression of gene regulatory elements in each cluster is shown in **Figure 3B**. The heatmaps of the differentially expressed gene regulatory elements in each cell of the three cell clusters are shown in **Figures 3C** and **D**.

### 3.4 Functional analysis of epithelial-mesenchymal transition

**Figure 4:**
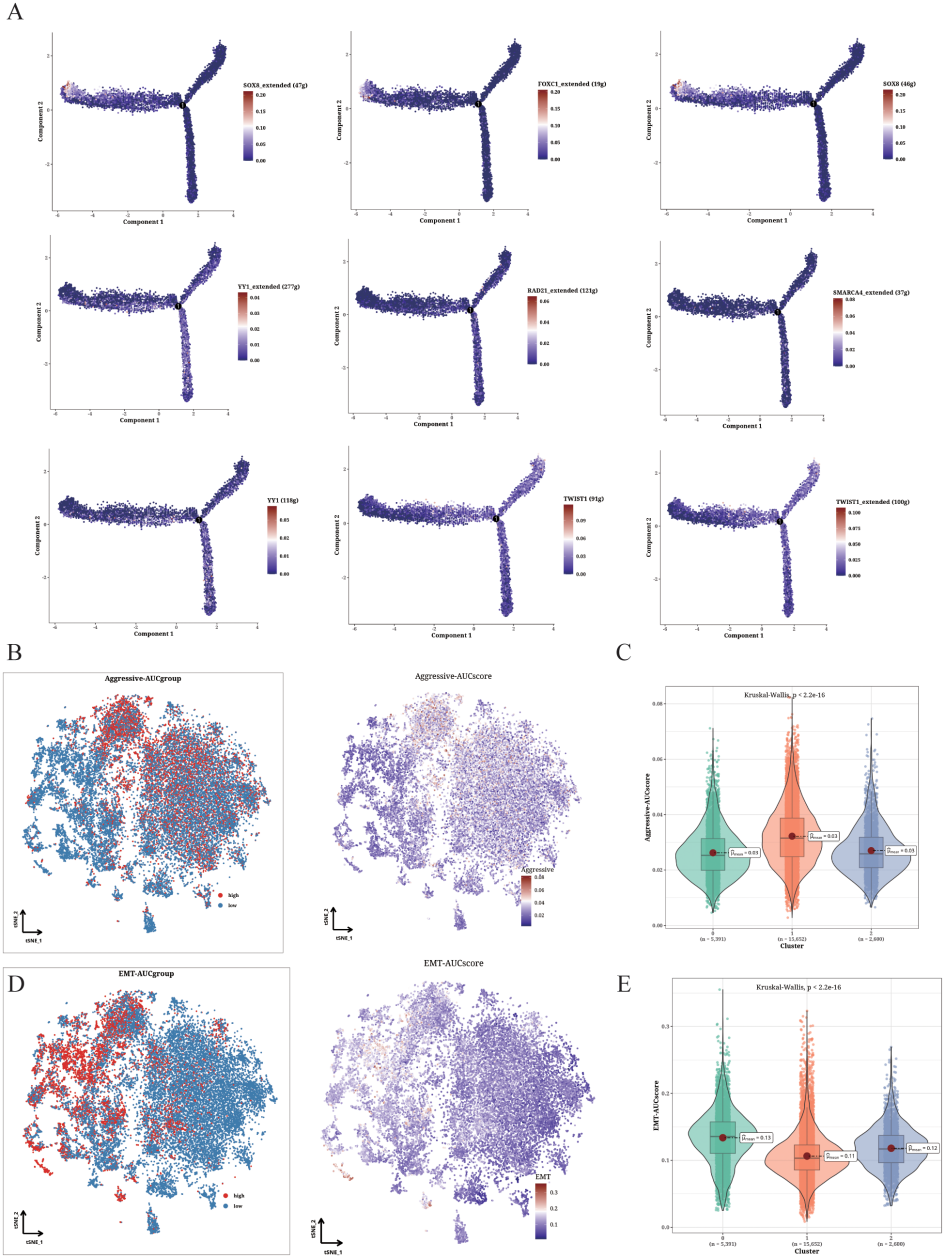
Functional analysis of Aggressive and EMT phenotypes. **(A)** Cell trajectory analysis showing the expression pattern of different identified TFs in various differentiation states. **(B, C)** Invasion levels of the three clusters shown in t-SNE plot **(B)** and violin plot **(C)**. **(D, E)** EMT levels of the three clusters displayed in t-SNE plot **(D)** and violin plot **(E)**.

The transcriptional factors with highest specificity for clusters 0-2 were integrated into the pseudo-time analysis (**Figure 4A**). FOXC1 and SOX8 were upregulated in cluster 0, RAD21, SMARCA4 and YY1 were upregulated in TMSB4X+ cells (cluster 1), and TWIST1 and YY1 were upregulated in SLPI+ cells (cluster 2). The TMSB4X+ cells displayed significantly higher scores for epithelial-mesenchymal transition (EMT), indicating enhanced invasion ability (**Figures 4B, C**). Additionally, as shown in **Figures 4D** and **E**, significant differences in EMT scores were observed between S100A1+ cells and TMSB4X+ and SLPI+ cells. Specifically, the EMT score of S100A1+ cells were significantly higher than that of TMSB4X+ and SLPI+ cells, suggesting that osteosarcoma cells in the TMSB4X+ cells exhibit a greater migration ability, possibly associated with an increased propensity for metastasis.

### 3.5 Correlation between OS cell clustering and prognosis

**Figure 5:**
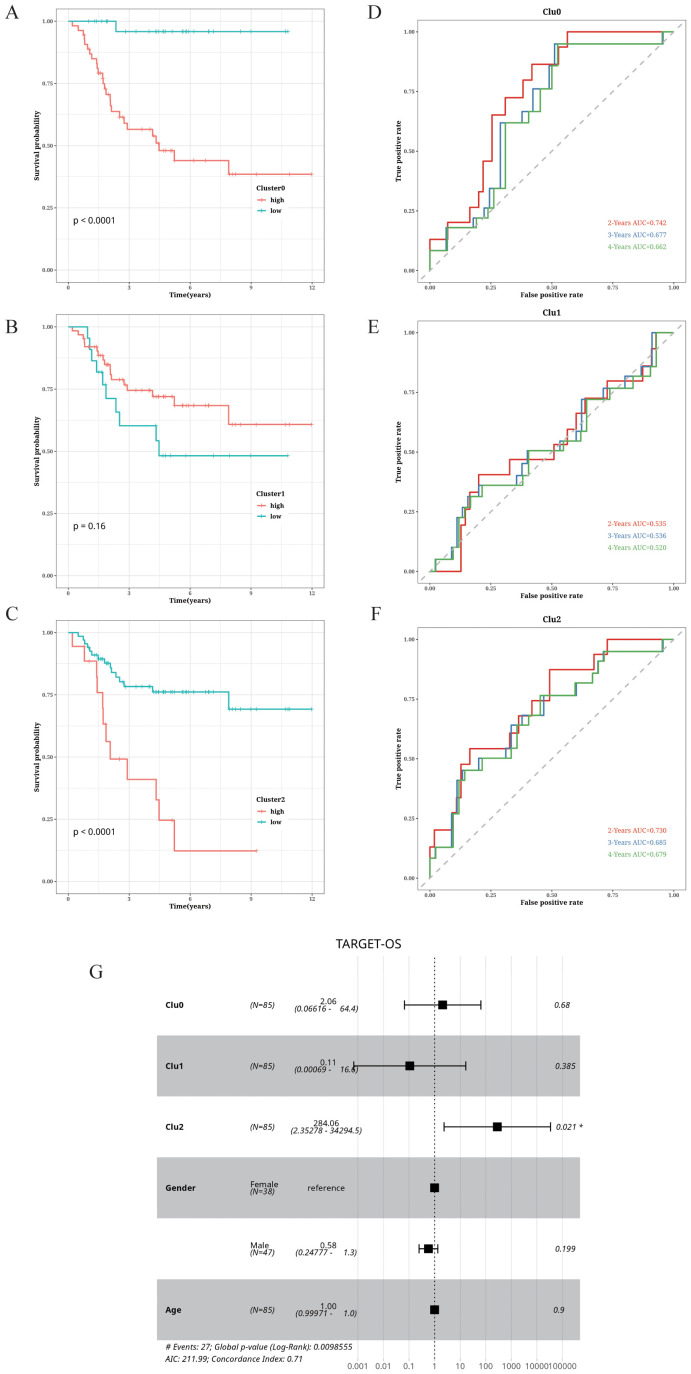
Correlation of cell cluster with prognosis in OS. **(A–C)** Impact of abundance of clusters 0, 1, and 2 on survival. **(D–F)** Time-dependent ROC curves of clusters 0, 1, and 2. **(G)** Forest plot showing the results of multifactor Cox analysis.

The prognostic relevance of OS cell clustering was determined by analyzing the survival rates of patients in the TARGET database. The patients with high abundance of clusters 0 and 2 displayed higher survival rates. In contrast, cluster 1 was associated with lower survival rates (**Figures 5A–C**). Additionally, we plotted the receiver operating characteristic (ROC) curves for 2-, 3-, and 4-year survival, and found that the area under the curve (AUC) for clusters 0 and 2 were above 0.65, indicating good predictive performance. Conversely, the AUC value for cluster 1 was relatively low (**Figures 5D–F**). To further assess the prognostic significance of the OS cell clusters, we performed a multivariate Cox analysis incorporating patient gender, age, cluster scores, and survival, and observed a significant correlation between the cluster 2 score and patient survival (**Figure 5G**).

### 3.6 Immune infiltration analysis

**Figure 6:**
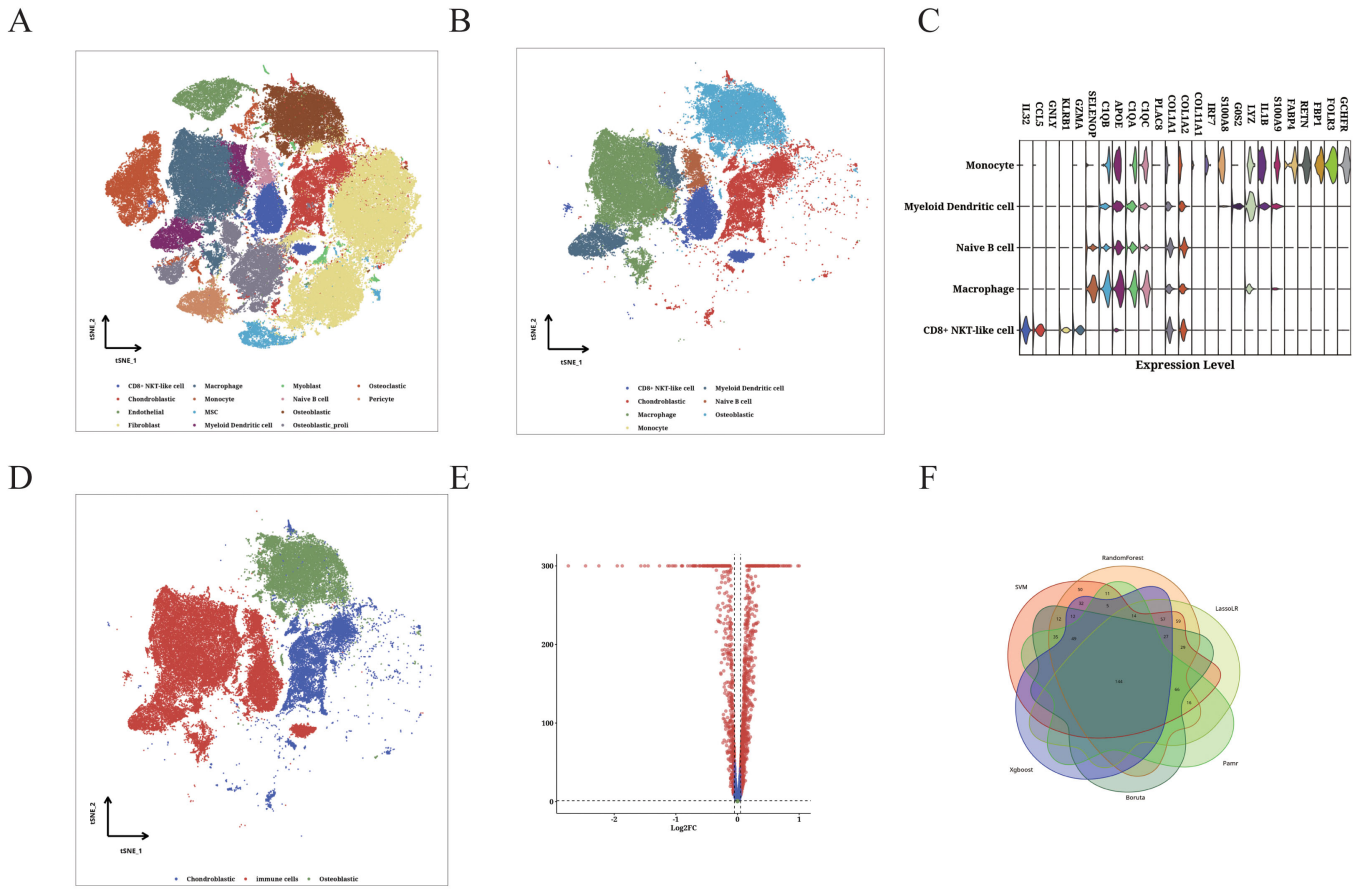
Identification of TIIC-RNA at single-cell level. **(A)** t-SNE plot of identified TME cells and OS cells. **(B)** t-SNE plot of identified OS cells and 5 types of immune cells. **(C)** Violin plot showing differentially expressed genes in the identified immune cells. **(D)** t-SNE plot of identified immune cells and OS cells. **(E)** Volcano plot displaying differentially expressed genes between immune cells and OS cells. **(F)** Venn diagram classifying intersecting genes identified by six ML algorithms.

Using the OS scRNA-seq dataset, we identified 12 microenvironment cells along with OS cells (**Figure 6A**). Further analysis focused on OS cells and 5 immune cells (**Figure 6B**). We identified the potential immune-related RNA (IURNA) for that cell type. By applying a TSI score threshold of less than 0.45, we further refined this list to identify IURNA specific to immune cells. To validate the accuracy of cell classification, we examined the DEGs in immune cells (**Figure 6C**). The t-SNE plot displayed the distribution of immune cells and OS cells (**Figure 6D**), and DEGs between immune cells and OS cells were calculated and presented in **Figure 6E**. The comparison identified 618 significantly up-regulated DEGs in immune cells, which were defined as TIIC-RNA. We employed six machine learning algorithms and identified 177 additional TIIC-RNAs based on previous TIIC-RNAs (**Figure 6F**).

### 3.7 Construction of the TIIC prognostic model

**Figure 7:**
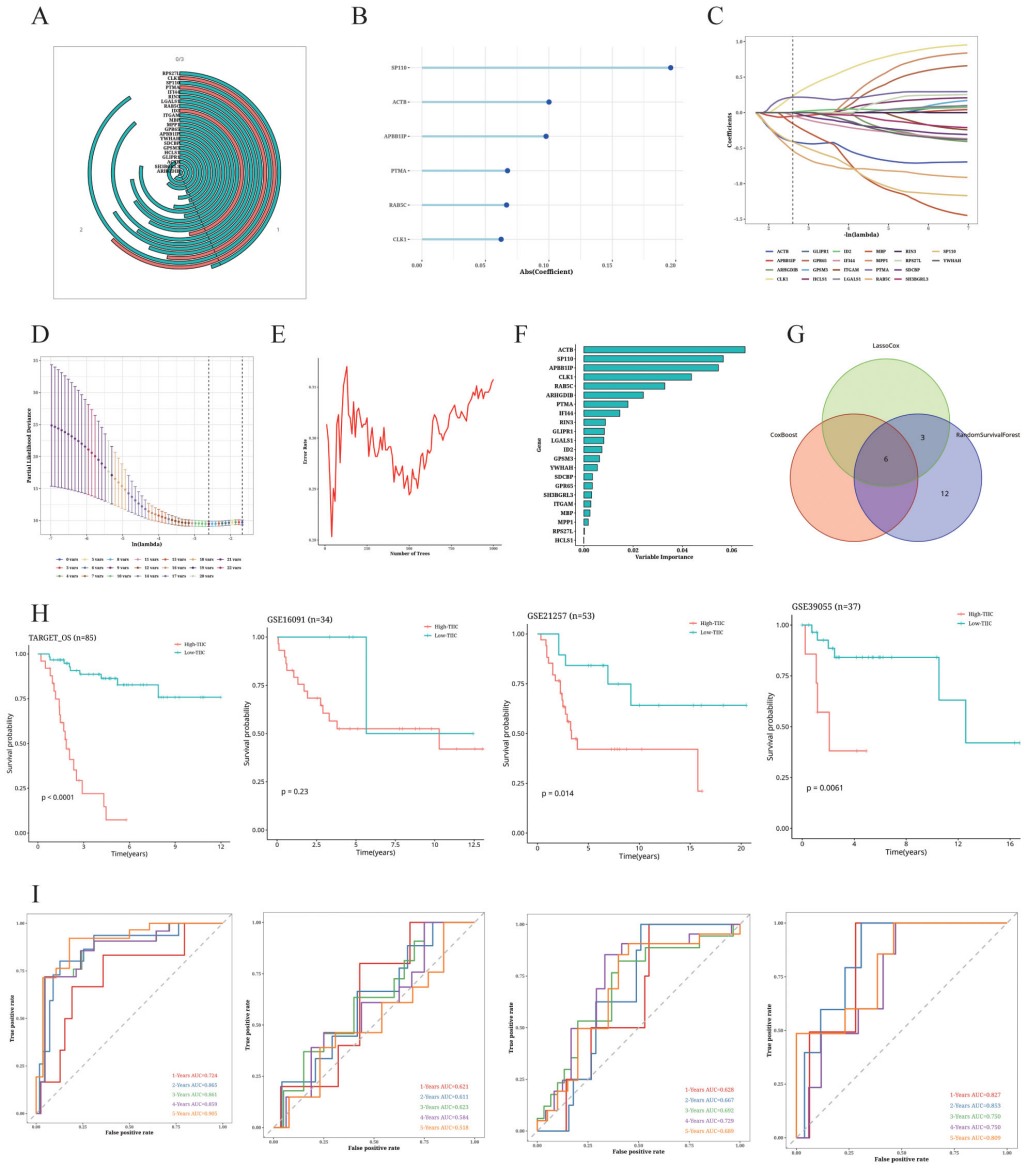
Construction of TIIC prognosis model. **(A)** Univariate Cox regression analysis of TIIC-related genes. **(B–F)** Dimension reduction of 22 prognostic genes using **(B)** CoxBoost algorithm, **(C, D)** LassoCox algorithm, and **(E, F)** random survival forest algorithm. **(G)** Venn diagram showing prognostic genes identified by all three ML algorithms. **(H)** Kaplan-Meier survival curves of OS patients of different TIIC feature scores in TARGET-OS and other validation datasets. **(I)** ROC curves of TIIC scores for predicting 1- to 5-year overall survival in the TARGET-OS and other validation datasets.

We identified 22 TIIC-RNAs in the TARGET dataset (**Figure 7A**), and screened the prognostic genes using CoxBoost (**Figure 7B**), LassoCox (**Figures 7C, D**), and Random Forest (**Figures 7E, F**) algorithms for intersection of mutual significant genes to determine the prognostic value of the TIIC-RNAs (**Figure 7G**). We used Venn diagrams to show the prognostic genes identified by all three ML algorithms (**Figure 7G**). The most reliable model was identified by calculating the C index using 20 ML algorithms, of which the Elastic Net (Enet) algorithm exhibited the highest scoring performance. The TIIC signature score was calculated from a panel of 20 prognostic TIIC-related RNAs. OS patients with higher TIIC scores showed poor outcomes in the TARGET-OS dataset as well as the validation datasets (**Figure 7H**). ROC curves of TIIC scores predicting 1-5 year overall survival in TARGET-OS and other validation datasets show that our model has good efficacy in the first five years (**Figure 7I**)

### 3.8 Comparison of the TIIC signature with other prognostic models

**Figure 8:**
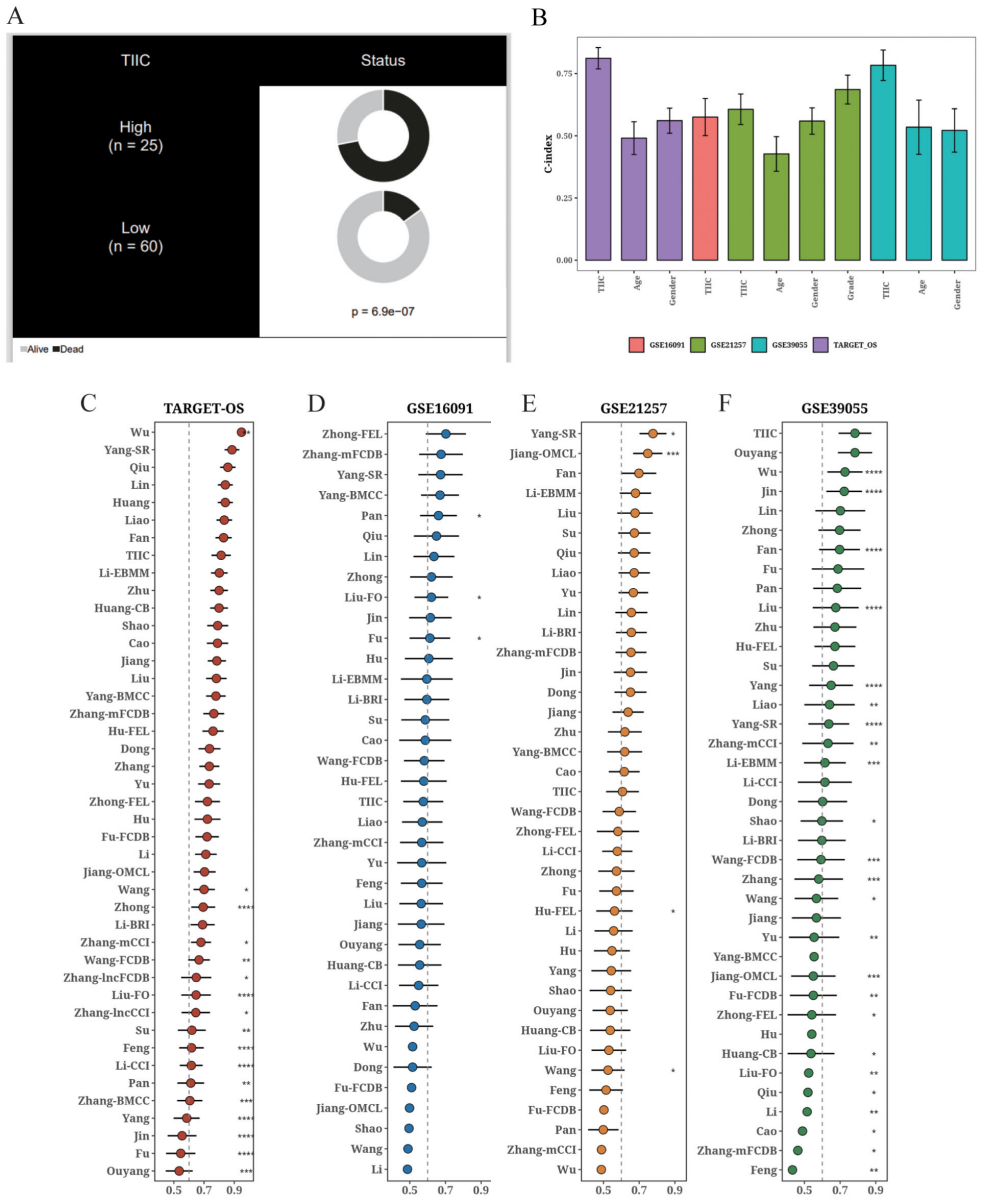
Comparison of the prognostic value of TIIC score and other prognostic models. **(A)** Circos plot showing different clinical factors in the TIIC-low and TICC-high groups. **(B)** C-index bar plot of TIIC score and various clinical factors in TARGET-OS and other validation datasets. **(C–F)** C-index plots of TIIC score and 42 prognostic models in TARGET-OS and other validation datasets.

The TIIC score correlated significantly with survival in the TARGET dataset (**Figure 8A**), and demonstrated higher C-index compared to age and gender (**Figure 8B**). We compared the C-index of the TIIC score with that of 42 prognostic models reported in literature, and found that the TIIC model outperformed most published models in the TARGET-OS and validation datasets (**Figures 8C–F**).

### 3.9 Putative biological mechanism of the TIIC model

**Figure 9:**
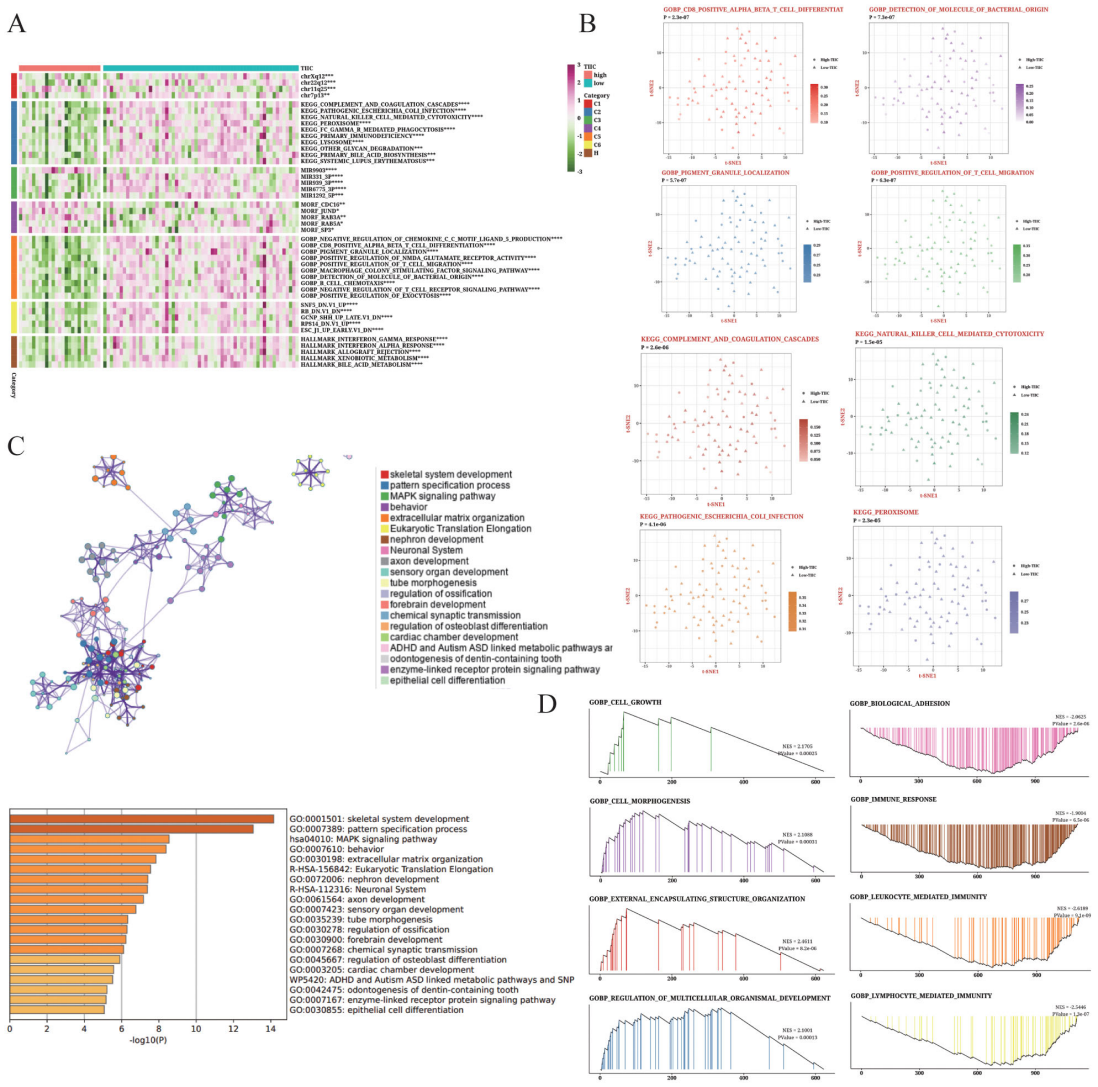
Biological characteristics of the TIIC signature in TARGET dataset. **(A)** Results of GSVA based on MsigDB showing the biological properties associated with TIIC score. **(B)** t-SNE plots illustrating the differences in GO terms and KEGG pathways between TIIC-low and TIIC-high groups. **(C)** Enrichment analysis of differentially expressed genes between the TIIC-low and TIIC-high groups based on Metascape. **(D)** GSEA results depicting the enrichment of GO and KEGG terms between the TIIC-high and TIIC-low groups.

The TIIC feature score showed a strong positive correlation with numerous pathways (**Figure 9A**), especially immune-related pathways including INF-gamma and alpha activation. We selected eight pathways from the GOBP and KEGG databases that exhibited significant differences between the two groups (**Figure 9B**). We also examined the enrichment results of up-regulated genes in the TIIC-high group using Metascape, which revealed their association with immune response and cell adhesion (**Figure 9C**). Moreover, GSEA of the dominant genes showed enrichment of cell growth and morphogenesis functions in the TIIC-high group (**Figure 9D**).

### 3.10 TIIC signature is significantly correlated with immune-related features

**Figure 10:**
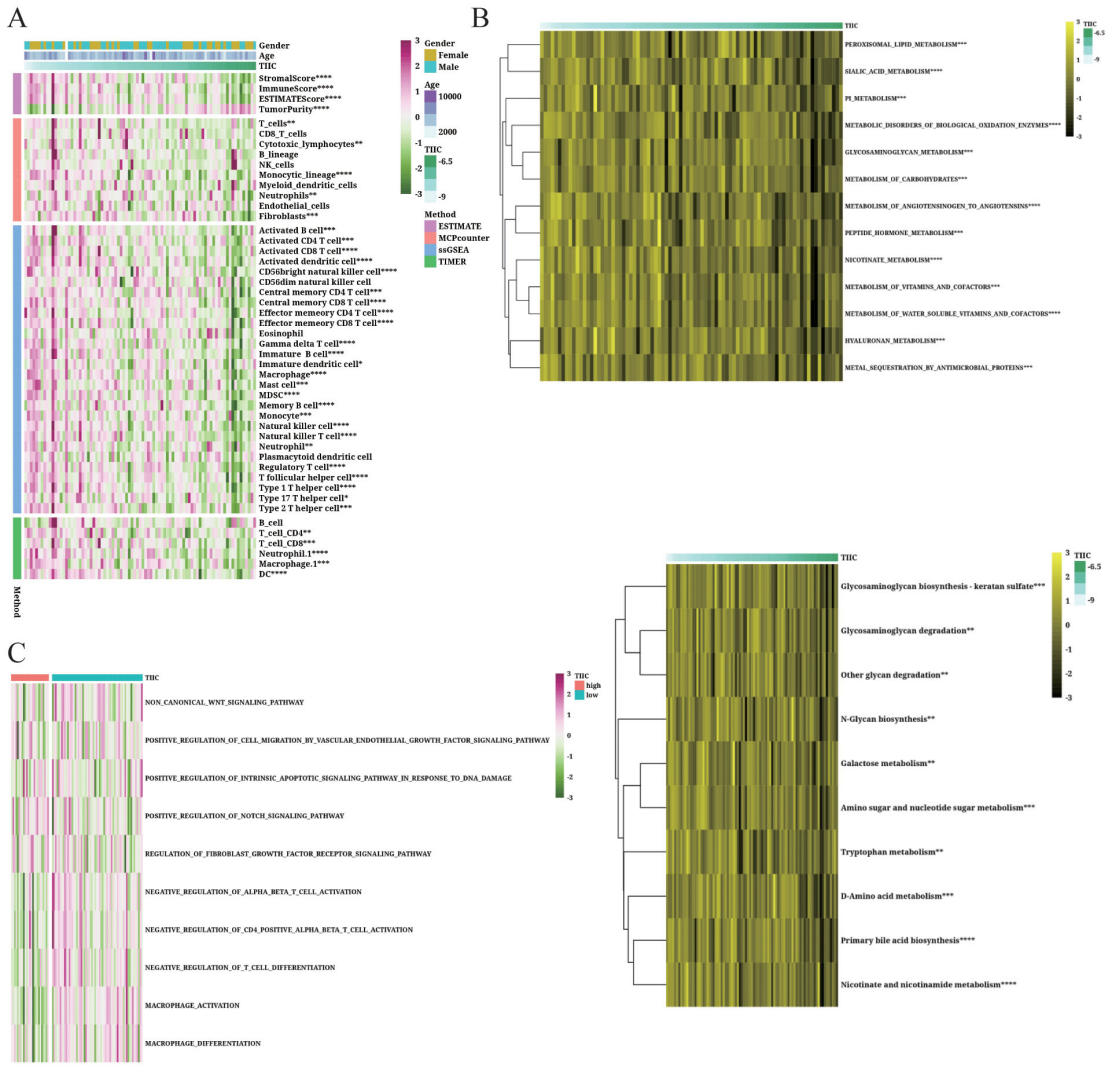
Immunological features of the TIIC signature in TARGET dataset. **(A, B)** Relationship between the TIIC score, immune infiltrating cells and immune regulatory genes. **(C)** Abundance of associated pathways in high TIIC group and low TIIC group.

The immune infiltrating cells and their activity were analyzed based on the TIIC score using the TIMER, ssGSEA, MCPcounter, and ESTIMATE algorithms. As shown in **Figure 10A**, the activity of most immune cells declined with the increase in TIIC score, especially that of CD8+ T cells and M1 macrophages, whereas the Tregs and MDSCs showed increased activity. We also compared the TIIC score with the enriched pathways in KEGG and reactome genes (**Figure 10B**) and determined the abundance of tumor-related pathways in the TIIC-high and TIIC-low groups (**Figure 10C**). Macrophage activation and differentiation were both lower in the TIIC-high group, which was consistent with former observation.

### 3.11 TIIC signature score can predict treatment response

**Figure 11:**
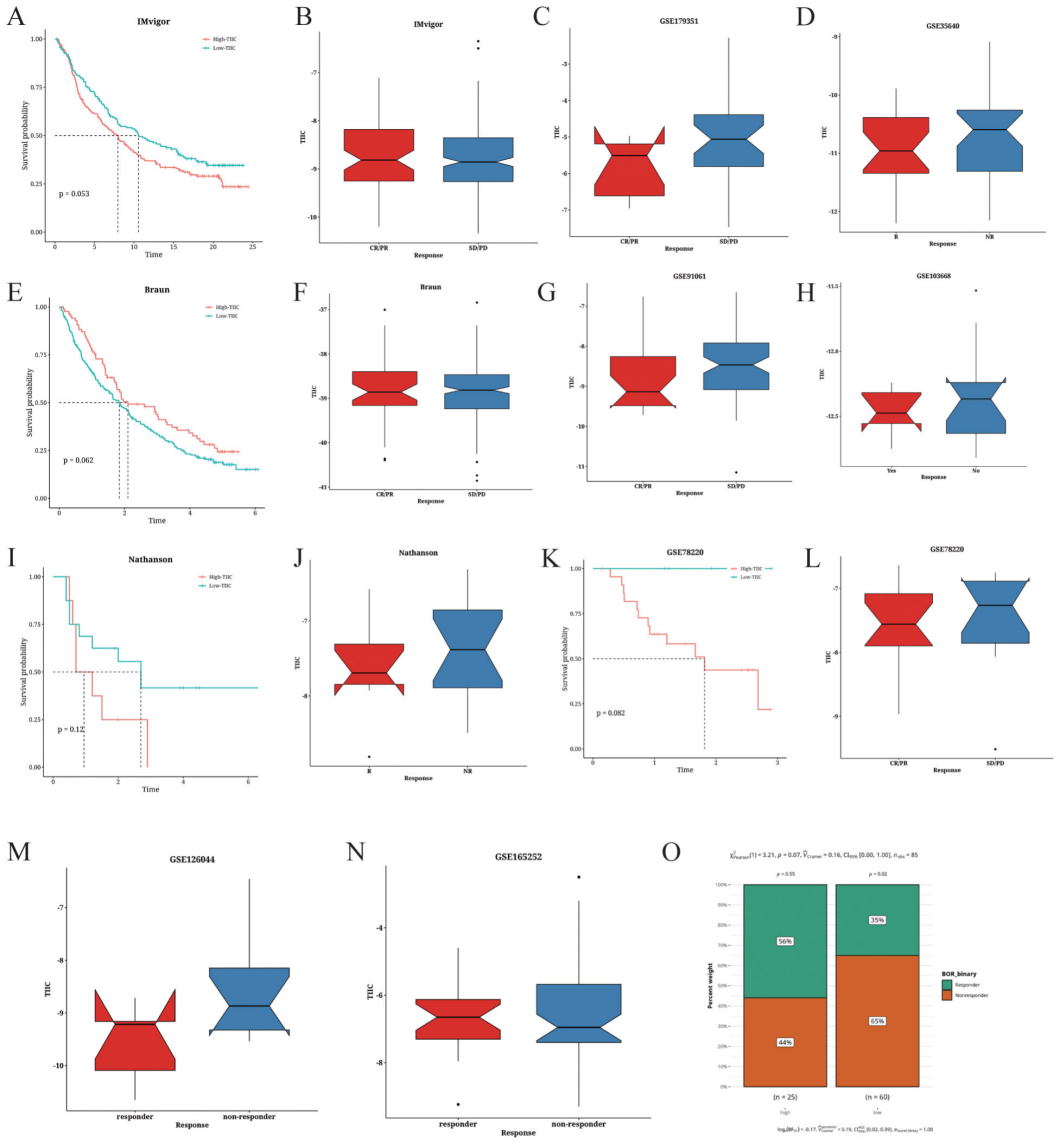
Prediction of the immunotherapeutic response based on TIIC signature scores. **(A)** Survival analysis of the IMvigor cohort based on TIIC scores. **(B–D)** Correlation between TIIC score and the immunotherapeutic response in the **(B)** IMvigor, **(C)** GSE179351, and **(D)** GSE35640 datasets. **(E)** Survival analysis of the Braun dataset based on TIIC scores. **(F–H)** Correlation between TIIC score and the immunotherapeutic response in the **(F)** Braun, **(G)** GSE91061, and **(H)** GSE103668 datasets. **(I)** Survival analysis of the Nathanson dataset based on TIIC scores. **(J)** Correlation between TIIC score and immune therapeutic response in the Nathanson dataset. **(K)** Survival analysis of the GSE78220 dataset based on TIIC scores. **(L–O)**. Correlation between TIIC score and immune therapeutic response in the **(L)** GSE78220, **(M)** GSE126044, **(N)** GSE165252, and **(O)** TARGET datasets.

The predictive value of the TIIC score for immunotherapy response was examined in various cancer datasets. As shown in **Figure 11A**, low TIIC scores correlated with better survival outcomes in patients with urothelial carcinoma (UC). Furthermore, UC patients with high TIIC scores demonstrated a better response to PD-L1 immunotherapy (**Figure 11B**). In the Braun dataset, renal cell carcinoma (RCC) patients with high TIIC feature scores exhibited improved survival outcomes (**Figure 11E**), while those with high TIIC scores responded better to PD-1 immunotherapy (**Figure 11F**). In the Nathanson dataset, low TIIC scores correlated with favorable prognosis (**Figure 11I**) as well as better response to immunotherapy (**Figure 11J**). Similar observations were made in the GSE78220 dataset (**Figures 11K, L**). Patients with high TIIC scores in the GSE165252 dataset demonstrated a better response to immunotherapy (**Figure 11N**). On the other hand, low TIIC scores were associated with better response to immunotherapy in the GSE179351 (COAD and PAAD) (**Figure 11C**), GSE35640 (**Figure 11D**), GSE126044 (**Figure 11M**), GSE91061 (**Figure 11G**), and GSE103668 (**Figure 11H**) datasets. Using the TIDE algorithm, we observed that the proportion of responders was relatively low in the TIIC-low group in the TARGET dataset (p=0.07, **Figure 11O**).

### 3.12 Prediction of metabolic characteristics associated with TIIC scores

**Figure 12:**
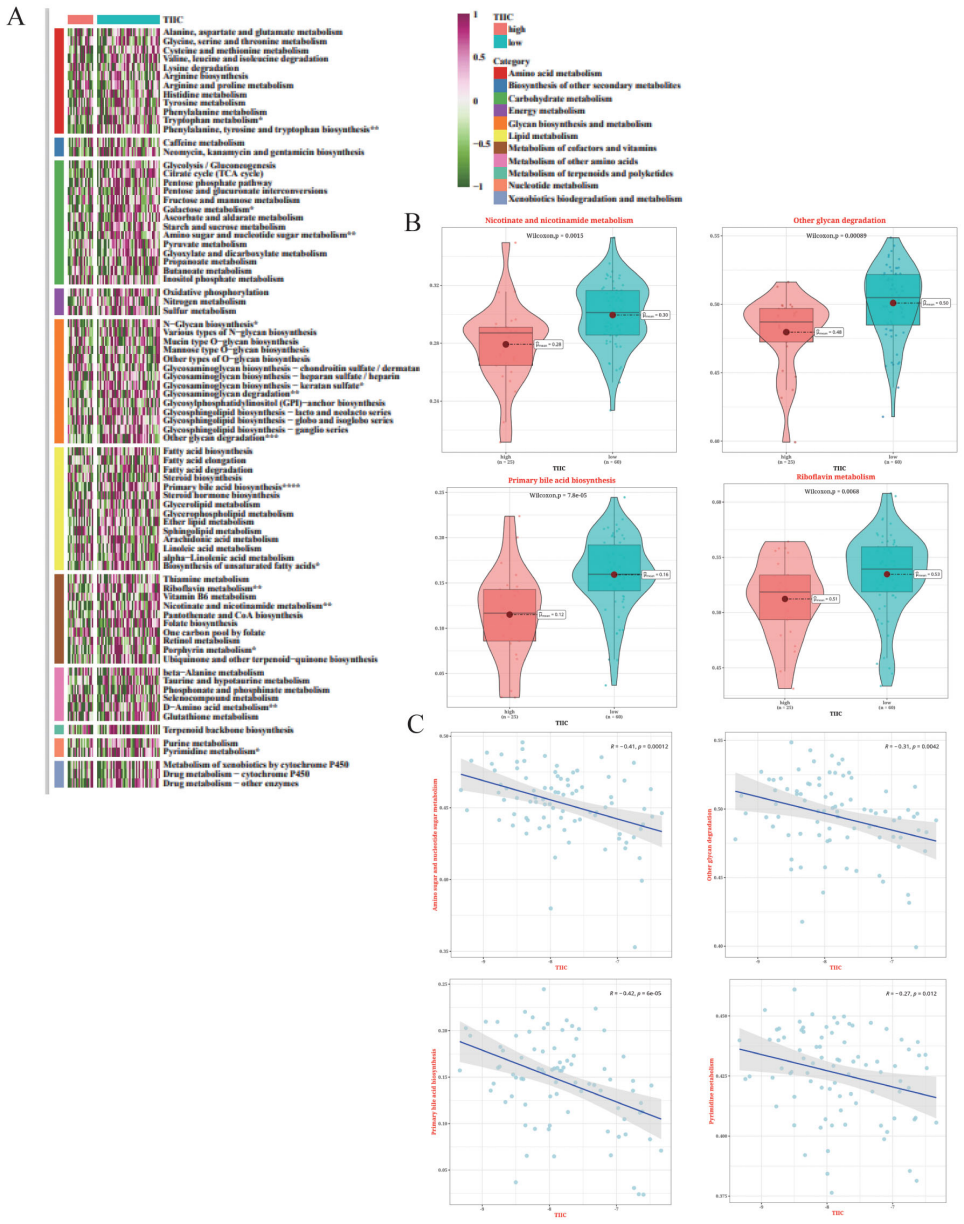
Metabolic characteristics associated with TIIC scores in the TARGET dataset. **(A)** Results of GSVA based on KEGG pathways for 11 metabolic categories in the TIIC score groups. **(B)** Differences in metabolic pathways between the TIIC-high and TIIC-low groups. **(C)** Correlation between TIIC feature scores and KEGG pathways in GSVA.

The metabolic characteristics associated with the TIIC signature were elucidated by GSVA on metabolic pathways from the KEGG database. The TIIC score was significantly correlated with several metabolic pathways (**Figure 12A**). Notably, riboflavin metabolism exhibited significantly higher activation rates in the TIIC-low group (**Figure 12B**). In addition, the TIIC score was negatively correlated with amino sugar and nucleotide sugar metabolism, and other glycan degradation (**Figure 12C**).

### 3.13 Analysis of SNV mutations and CNV differences

**Figure 13:**
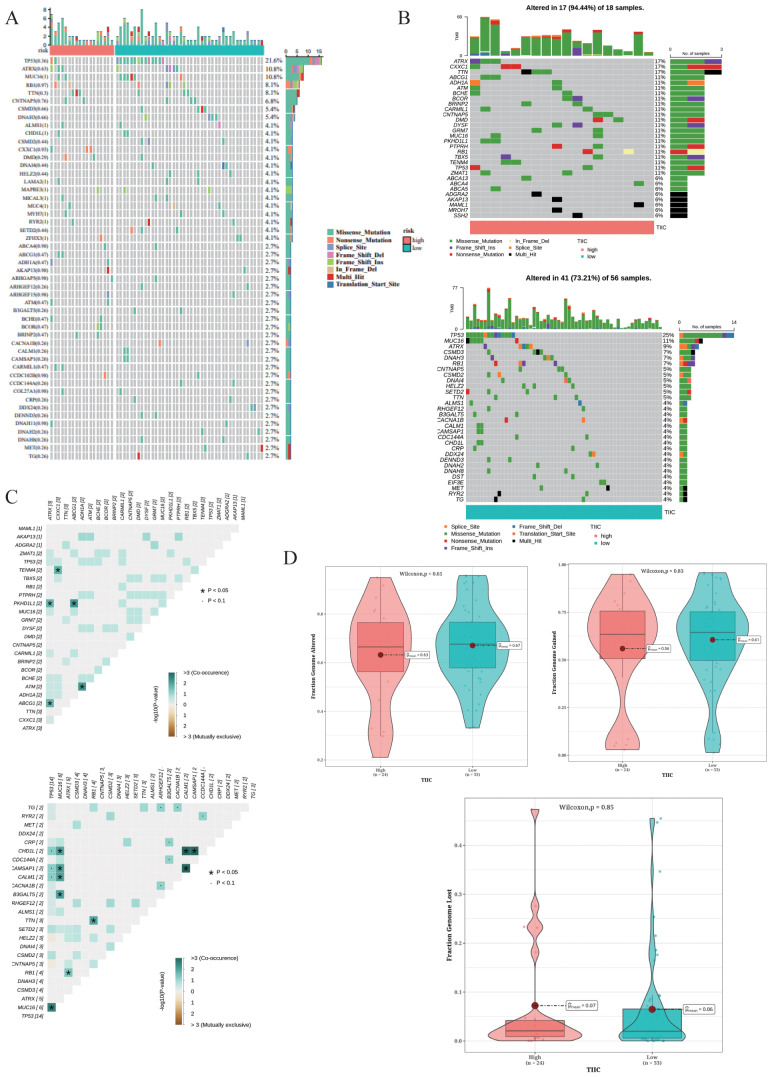
Mutation landscape in the TARGET dataset. **(A)** Waterfall plot of the top 50 mutated genes in the TARGET dataset. **(B)** Mutation landscapes of OS patients grouped by TIIC score. **(C)** Exclusive and co-occurring mutations in the OS patients with different TIIC scores. **(D)** Distribution of CNVs in the OS patients stratified by TIIC score, with FGA, FGG, and FGL as features.

The top 50 mutated genes in the two risk groups are shown in the waterfall diagram in **Figure 13A**. We observed higher mutation rates in TP53 (21.6%), ATRX (10.8%), and MUC16 (10.8%) (**Figure 13A**). The TIIC-high group showed a higher frequency of mutations in ATRX, CXXC1, and TTN, while TP53, MUC16, and ATRX were the predominantly mutated genes in the TIIC-low group (**Figures 13B, C**). The TIIC-high group also exhibited higher chromosomal instability, characterized by FGA, although statistical significance was not observed (**Figure 13D**).

### 3.14 CLK1 promotes the proliferation and migration of OS cells

**Figure 14:**
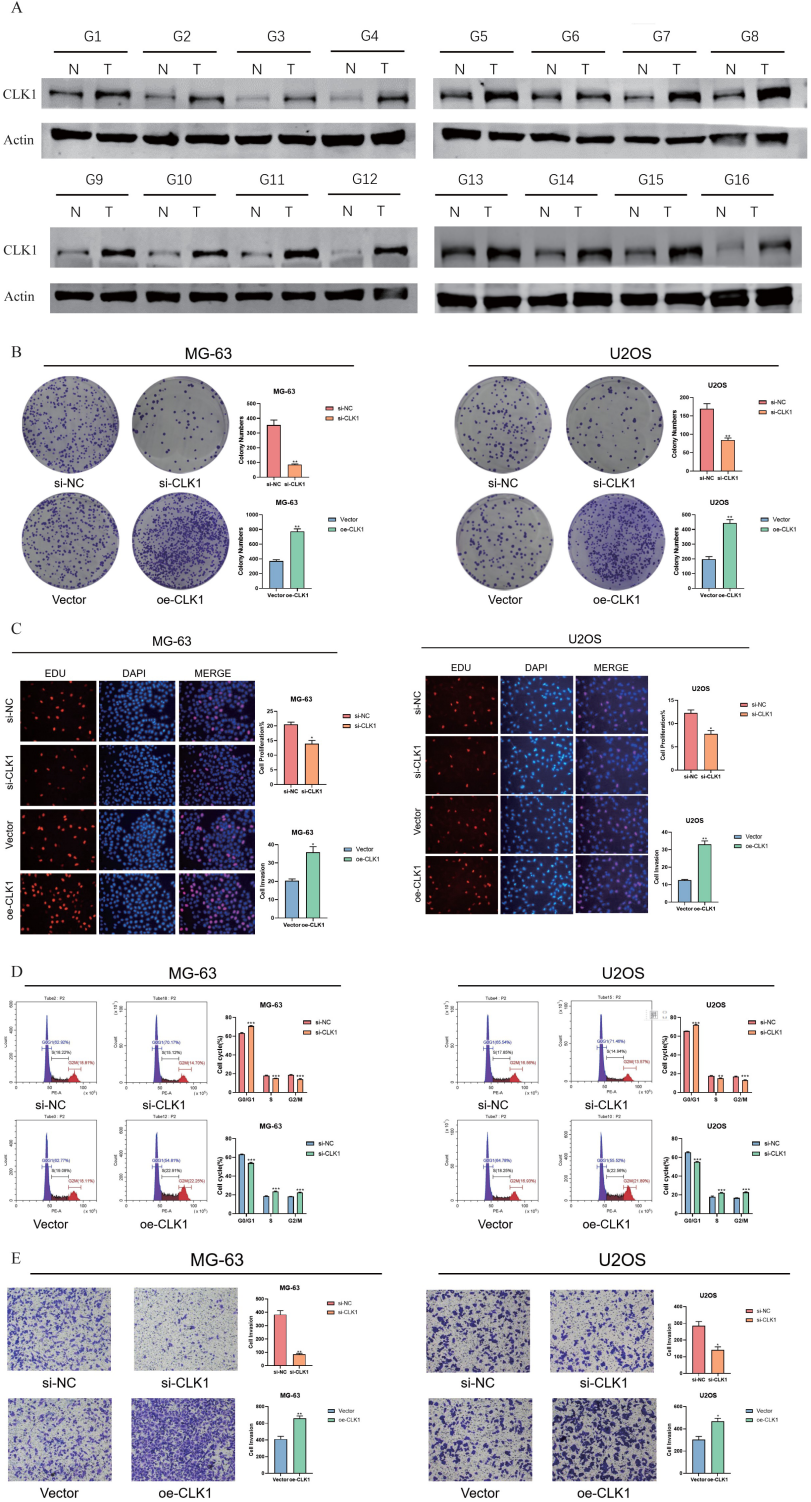
CLK1 promotes OS proliferation and migration. **(A)** Immunoblot showing CLK1 protein expression in the OS tissues. **(B)** Colonies formed by the CLK1-overexpressing and CLK1-knockdown MG63 and U2OS cell lines. **(C)** The up-regulation and down-regulation of EDU staining of the MG63 and U2OS cell lines under the condition of CLK1 overexpression and knockout, reflecting the proliferating capability of the OS cells. **(D)** Flow cytometry assessing the MG63 and U2OS cell lines in the phase of G2/M state and corresponding quantification results. **(E)** Transwell analysis assessing the migration of the MG63 and U2OS cell lines under the condition of CLK1 overexpression and knockout and the corresponding quantification results. (*, denotes a significance level of p < 0.05; **, p < 0.01; ***, p < 0.001).

The functional role of CLK1 was further investigated through a series of *in vitro* experiments. The CLK1 protein was significantly upregulated in the OS tissues, highlighting its potential as an oncogene (**Figure 14A**). Knocking down CLK1 led to a significant loss in the clonogenic potential of the OS cells, as indicated by the decrease in the number of colonies (**Figure 14B**). Conversely, the overexpression of CLK1 increased their colony-forming capacity, suggesting that CLK1 is necessary for the growth of OS cells. Consistent with this, the MG63 and U2OS cell lines exhibited higher EDU incorporation upon CLK1 overexpression, while CLK1 knockdown decreased the proportion of EDU+ proliferating cells (**Figure 14C**). Furthermore, loss of CLK1 decreased the proportion of OS cells in the G2/M state of the cell cycle (**Figure 14D**). CLK1 overexpression also promoted the migration of MG63 and U2OS cells in the transwell assay, while CLK1 knockout resulted in a decrease in migration capacity (**Figure 14E**). Overall, these findings provide mechanistic insights into the role of CLK1 in promoting OS proliferation and migration, emphasizing its potential as a therapeutic target.

## 4 Discussion

In this study, we examined the genomic and transcriptional heterogeneity of OS cells and their interactions with other cells in the TME. The OS cells had higher CNVs compared to endothelial cells, indicating genetic instability. We also identified distinct transcriptional subtypes within the OS cells, of which cluster 1 showed characteristics of terminal differentiation. Furthermore, the expression levels of S100A1, TMSB4X, and SLPI were significantly altered with the pseudo-time trajectory of the cells. Functional analysis showed that cluster 1 cells exhibited greater aggressiveness and correlated with worse clinical outcomes. We developed a prognostic model based on TIIC-related genes using machine learning, and found that higher TIIC signature scores were associated with lower infiltration of cytotoxic immune cells and inferior immune response in multiple OS datasets. We also validated the TIIC model in cancer datasets, and found that lower scores were associated with superior immune response and survival rates. This suggests that the immune landscape in the TME could predict prognosis and response to immunotherapy in OS patients.

S100A1, a calcium-binding protein, was upregulated along the pseudo-time progression. Moreover, the S100A1+ tumor cells exhibited active communication with other cells. S100A1 is overexpressed in ovarian cancer tissues, and is associated with lymph node metastasis, FIGO stages, and tumor grades. Furthermore, *in vitro* experiments have shown that S100A1 promotes the proliferation and migration of ovarian cancer cells (16). Likewise, S100A1 is significantly upregulated in papillary thyroid carcinoma (PTC) tissues, and correlates with tumor size and lymph node metastasis. Silencing S100A1 in PTC cells inhibited their proliferation and migration via the Hippo/YAP pathway (17). Collectively, these findings suggest that S100A1 is a pan-cancer oncogene and a promising diagnostic and prognostic biomarker for various tumors. However, the role of S100A1 in the genesis and progression of OS remains to be elucidated.

CLK1 is a Cdc2-like kinase that was identified as a crucial risk factor in our TIIC-based model. Knocking down CLK1 in the OS cell lines inhibited their proliferation, invasion, and migration by decreasing phosphorylation of SRSF2. Experiments using patient-derived tumor samples have shown that CLK1 is a potential target for gastric cancer treatment (18). Furthermore, knockdown of CLK1 In glioma cells (GL261) increased aerobic glycolysis and expression of HIF-1α via the AMPK/mTOR signaling pathway (19). Thus, CLK1 warrants further investigation as a promising target for treating OS, although no studies have characterized the underlying molecular mechanisms so far.

Despite the introduction of neoadjuvant chemotherapy, the rates recurrence and metastasis remain high in OS patients (20). B7-H1/PD-1 is a crucial immune checkpoint in OS and other pediatric solid tumors. Previous studies have indicated that B7-H1/PD-1 blockade monotherapy is less effective and can lead to numerous adverse reactions in OS patients (21–23). On the other hand, combination of PD-1 blockade with other therapies has demonstrated more favorable outcomes for OS in cellular and animal models (24). The TIIC signature score established in our study displayed satisfactory efficacy in predicting the immune response across multiple cohorts, and could be integrated into clinical practice.

## 5 Conclusion

We developed a TIIC signature to predict the prognosis and immunotherapy response in OS patients. The TIIC score effectively stratified OS patients based on prognostic outcomes, and was significantly associated with immune infiltration and immune response. Moreover, CLK1 is a potential oncogenic factor in OS development and a potential therapeutic target.

## Data Availability

The original contributions presented in the study are publicly available. This data can be found here GEO database (GSE16091, GSE21257, and GSE39055), and TARGET database (https://ocg.cancer.gov/programs/target).
